# Awareness, Attitudes, Prevention, and Perceptions of COVID-19 Outbreak among Nurses in Saudi Arabia

**DOI:** 10.3390/ijerph17218269

**Published:** 2020-11-09

**Authors:** Reem Al-Dossary, Majed Alamri, Hamdan Albaqawi, Khaled Al Hosis, Mohammed Aljeldah, Mohammed Aljohan, Khalid Aljohani, Noura Almadani, Bader Alrasheadi, Rawaih Falatah, Joseph Almazan

**Affiliations:** 1 Nursing Education Department Nursing College, Imam Abdulrahman bin Faisal University, Dammam 34221, Saudi Arabia; rnaldosari@iau.edu.sa; 2 Nursing Department, College of Applied Medical Sciences, University of Hafr Al Batin, Hafr Al Batin 39911, Saudi Arabia; m.alamri@uhb.edu.sa; 3 College of Nursing, University of Hail, Hail 81491, Saudi Arabia; h.albaqawi@uoh.edu.sa; 4 Department of Nursing Education, Nursing College, Qassim University, Buraidah 51452, Saudi Arabia; 3747@qu.edu.sa; 5 Clinical Laboratory Science Department, College of Applied Medical Sciences, University of Hafr Al Batin, Hafr Al Batin 39911, Saudi Arabia; mmaljeldah@uhb.edu.sa; 6 Nursing College, Taibah University, Al-Madinah 42362, Saudi Arabia; msejohani@taibahu.edu.sa; 7 Community Health Nursing, Department, Nursing College Taibah University, Al-Madinah 42356, Saudi Arabia; kajohani@taibahu.edu.sa; 8 Nursing Management & Education Department, Nursing College, Princess Nourah Bint Abdulrahman University, Riyadh 84428, Saudi Arabia; naalmadani@pnu.edu.sa; 9 Nursing Department, College of Applied Medical Sciences, Majmaah University, Majmaah 11952, Saudi Arabia; b.alrasheadi@mu.edu.sa; 10 Nursing Administration & Education Department, College of Nursing, King Saud University, Riyadh 13253, Saudi Arabia; rfalatah@ksu.edu.sa; 11 School of Medicine, Nazarbayev University, Nursultan 010000, Kazakhstan

**Keywords:** COVID-19, nurses, outbreak, pandemic, Saudi Arabia

## Abstract

The newly discovered coronavirus (COVID-19) has become a pandemic, infecting thousands of people around the world. This study examines nurses’ demographic information (age, gender, marital status, area of practice, total years of experience in the current hospital, work region, monthly salary, educational level, workplace, nationality, working hours per day, total nursing experience, and the respondents’ main source of information on COVID-19), awareness, attitudes, prevention, and perceptions of COVID-19 during the outbreak in Saudi Arabia. A cross-sectional descriptive design of 500 nurses working at government and non-governmental hospitals in five regions in Saudi Arabia were selected using convenience sampling. The Kruskal–Wallis test was applied and the Mann–Whitney test was utilized as a post hoc test. The majority of nurses in this study, 96.85%, had excellent knowledge of COVID-19. Some (83.2%) of nurses reported significant prevention knowledge and treatment skills about COVID-19, while 7.6% had little knowledge about prevention. More than half of the nurses (60.4%) had high positive attitudes toward caring for COVID-19 patients. In conclusion, female nurses, married nurses, and bachelor’s degree nurses had greater awareness, better attitude, and prevention clinical experience towards COVID-19. Meanwhile, non-Saudi nurses had higher self-reported awareness, positive attitudes, optimal prevention, and positive perceptions compared to Saudi nurses. This study provides baseline information immediately needed to enable health authorities to prioritize training programs that support nurses during the COVID-19 pandemic.

## 1. Introduction

The newly discovered coronavirus (COVID-19) (2019-nCoV) was found in December 2019, which emerged in Wuhan, China, and has infected millions of people worldwide [1]. Coronavirus is a communicable disease deriving from a large family of viruses that causes the illness [1]. It is similar to Middle East Respiratory Syndrome (MERS-CoV) and Severe Acute Respiratory Syndrome (SARS-CoV), with typical symptoms such as fever, shortness of breath, cough, and acute respiratory failure [2]. It is also complicated by the fact that patients may present with atypical symptoms. This means COVID-19 has clinical presentations ranging from asymptomatic to acute respiratory distress syndrome, and multiple-organ failure that leads to death [2]. In addition, COVID-19 can be transmitted from person to person through respiratory droplets [3]. On January 30, 2020, COVID-19 was announced as a pandemic public health emergency by the World Health Organization [4], and recently, cases of the virus surpassed more than 3 million worldwide with increasing mortality [5].

As of 2 November 2020, countries most affected by this outbreak are the United States (9,208,876 cases), India (8,229,313), and Brazil (5,545,705) [5]. In recent months, the COVID-19 infection has caused the unparalleled hastening of infection transmission worldwide, mostly affecting healthcare workers’ well-being. For example, one recent study in Poland found that healthcare workers who are exposed to COVID-19 infection have greater risk of depression, anxiety, and sleep disorders [6]. Similar results were also found in one study in Iran [7] and China [8], finding that nurses experience greater stress, extreme physical fatigue, anxiety, and insomnia during rendering of care, leading to reduced quality of patient care. In another study in Greece, nurses and doctors working in public hospitals had issues of sleep disorders during the pandemic [9]. In one of the studies, in Spain, healthcare workers reported that they were overwhelmed about their workload due to the increased number of COVID-19 patients admitted in the hospitals [10]. One of the studies in Pakistan, regarding the mental health of nurses, discovered that healthcare workers’ COVID-19 exposure has a substantial influence on their trauma, psychological distress, and turnover intention [11].

In Saudi Arabia, the first positive case was reported by the Ministry of Health (MOH) on 2 March 2020, and the number doubled in the country within one month, representing a critical challenge for healthcare professionals [12]. With 347,656 registered cases (as of 3 November 2020) in the country [13], the Saudi government has focused on preparedness by undertaking unprecedented precautionary measures [7], such as widespread quarantine use, airport and border surveillance and lockdown, and intensive infection control training for healthcare professionals [14]. Knowing that those working in hospitals are at higher risk of secondary infection or spreading the virus to colleagues, family, and friends [15], nurses should have awareness and knowledge of the disease and infection control measures to prevent spread. However, empirical data reports COVID-19 is challenging to nurses due to the novelty of the disease, the lack of information, training, and seminars to care for patients with the virus, and the psychological trauma resulting from patient deaths. Since it is such a new infection, misunderstanding COVID-19 signs and symptoms and incorrect treatment by nurses might speed the spread of hospital infection [16].

In fact, there is limited information regarding nurses’ knowledge, attitudes, perceptions, and knowledge about the COVID-19 outbreak. Thus, it is crucial to understand what nurses know about the virus, and their knowledge of pandemic complications and disease prevention. Further, Paitiraki et al. [17] pointed out that nurses’ perceptions change as the patient suffers from the disease. Nurses may have varied perceptions of COVID-19, and therefore may apply different clinical management strategies, leading to different outcomes. Nurses need to develop a solid foundation of the disease process to play a greater role in disease control. How nurses perceive and respond to COVID-19 is critical to expedite positive outcomes. Another contributing factor that affects individual perception about the disease is their attitude toward the disease [18].

In Saudi Arabia, there is no empirical data yet regarding nurses’ knowledge, attitudes, and disease perception. Since nurses, as first-line providers, are in close interaction with patients with COVID-19, they are a critical part of the disease transmission/care chain. If knowledge is power, the more one knows about risk factors, how to acquire knowledge, prevention practices, attitudes, and perceptions of nurses towards COVID-19 outbreak in the country, the better to break the transmission chain. In fact, given the increase in COVID-19 cases in Saudi Arabia and the need to expedite the country’s pandemic outbreak management, it is crucial to understand how nurses perceive the virus at this critical moment. This baseline information is needed for coordination and planning of care to ensure maximum function of the healthcare system, effective dissemination of accurate and timely information, and improving the well-being of nurses in Saudi health services—critical strategies for maximizing nurses’ health in future crises.

Finally, the results of the study could provide health authorities with information to enable them to prioritize training and other activities aimed at effectively improving nurses’ well-being and hence the quality of their care delivery. The purpose of this study is to examine nurses’ awareness, prevention, attitudes, and perceptions during the COVID-19 outbreak in Saudi Arabia.

## 2. Methods

### 2.1. Design

A cross-sectional descriptive design was employed using a self-report questionnaire in this study.

### 2.2. Sampling and Data Collection

Nurses working at government and non-governmental hospitals in five regions in Saudi Arabia were selected using convenience sampling. These hospitals were all tertiary care facilities treating COVID-19 patients, and each had a bed capacity of 300 to 400 patients. The inclusion criteria were the following: (a) being a registered nurse at the Saudi Commission for Health Specialties (SCFHS), (b) working as a nurse in the current hospital for the past six months, (c) dealing with COVID-19 patients, and (d) consenting to participate in the study.

This study was carried out after receiving approval from the Ethics Review Committee of Ministry of Health Hafr Al-batin (approval number 20; KACTS No.H-05-FT-083). Informed consent was implied to participate in the survey, information confidentiality was guaranteed, and answers were treated with the strictest confidence. Each survey also included a cover letter explaining the study purpose, voluntary participation, and that participants have the right to withdraw any time.

Data collection was done through an online questionnaire, from April to May 2020, which took about 20 min to complete. After completing the survey, answers were automatically tallied in an Excel table in a Google form document. Initially, 624 nurses conveniently selected and consented to participate. However, 500 nurses completed the survey (response rate = 80.12 %).

Finally, during this data collection period (24 May 2020), there were 70,161 confirmed COVID-19 cases, with 379 deaths in the country [19].

### 2.3. Questionnaire

The survey was developed by the researchers, based on Saudi Ministry of Health [14] and World Health Organization (WHO) guidelines [2,3].

This questionnaire assessed nurses’ awareness about COVID-19 in terms of the causative virus, signs and symptoms, transmission, and management protocol. The survey also enabled individual nurses to determine the complications, preventive methods, attitudes, perceptions, and practices which served as baseline information needed to improve patient care (Please see Supplementary Materials).

The questionnaire was composed of five domains:

Demographic and work-related characteristics. Part one of the questionnaire included the nurses’ demographic information, such as age, gender, marital status, area of practice, total years of experience in the current hospital, work region, monthly salary, educational level, workplace, nationality, working hours per day, total years of experience as a nurse, and the respondents’ main source of information on COVID-19.

Awareness. Awareness referred to information acquired through experience or education. This study asked what the respondent knew about COVID-19 and its route of transmission, how it infects another person, and how the respondent believes it should be treated. Further, it assessed awareness about COVID-19 in terms of causation, signs and symptoms, transmission, management protocol, complications, and preventive methods. It contained 38 statements with a three-point Likert scale (1 = yes to 3 = don’t know). An overall summation score of 38 meant higher self-reported awareness. A mean score was generated to determine the knowledge level for participants. Knowledge level was classified into five levels: “No knowledge” for those who answered yes to less than eight questions; “Some knowledge” for those who answered yes to at least 8 but less than 15 questions; “Good knowledge” for those who answered yes to at least 15 but less than 23 questions; “Very good knowledge” for those who answered yes to at least 23 but less than 30 questions; and “Excellent knowledge” for those who answered yes to at least 30 but less than 38 questions.

Prevention. Conceptually, prevention refers to the act of hindering or preventing. In this study, it referred to the preventive practice (knowledge and skills) of nurses in dealing with COVID-19. This section contained 14 statements with a five-point Likert scale (1 = Strongly Disagree to 5 = Strongly Agree). A mean score was generated to determine the prevention domain with five levels: “Lowest prevention” for answers with a mean value between 1–1.79; “Low prevention” for answers with a mean value between 1.8–2.59; “Average prevention” for answers with a mean value between 2.6–3.39; “High prevention” for answers with a mean value between 3.4–4.19; and “Highest prevention” for answers with a mean value between 4.2–5.

Attitude. Attitude is described as a feeling or opinion about something or someone. In this study, it was operationally defined as a predisposition of nurses to respond positively or negatively towards providing care to COVID-19 patients. Part three of the survey assessed nurses’ willingness to provide care to diagnosed patients and discussed their feelings about acquiring the infection and transmitting it to their families. It contained three statements with a three-point Likert scale (1 = yes to 3 = don’t know). An overall summation score was computed for the entire scale, with a score of fewer than three indicating a low attitude. A mean score was generated to determine the attitude domain with five levels as: “Very low attitude” for answers with a mean value between 1–1.39; “Low attitude” for answers with a mean value between 1.4–1.79; “Average attitude” for answers with a mean value between 1.8–2.19; “Good attitude” for answers with a mean value between 2.2–2.59; and “Excellent attitude” for answers with a mean value between 2.6–3.

Perception. Perception is how something is regarded, understood, or interpreted. For the purposes of this study, it was defined as how nurses take information on COVID-19 and respond to it. This section assessed nurses’ perceptions about their awareness, clinical experience, the availability of COVID-19 care protocols at their hospital, and if they received training from their hospitals. It contained four statements with a three-point Likert scale (1 = yes to 3 = don’t know). An overall summation score was computed for the entire scale; a score of less than four meant less self-reported perception. A mean score was generated to determine the perception domain with five levels: “Very low perception” where there was a mean value between 1–1.39; “Low perception” when answers had a mean value between 1.4–1.79; “Average perception” when answers had a mean value between 1.8–2.19; “High perception” for answers with a mean value between 2.2–2.59; and “Very high perception” for answers with a mean value between 2.6–3.

### 2.4. Validity and Reliability of the Questionnaire

To begin, the tool was sent to a panel of experts (three assistant professors, one professor, and three associate professors) for face and internal content validity. It was evaluated comprehensively based on clear aims and objectives, well-designed content (demographic, awareness, prevention, attitudes, and perception), the specified target group, and appropriate use of language. Then, it was pilot tested on 30 nurses in different settings with the same characteristics of the target respondents.

The reliability test was applied for each domain due to the differences in the measured dimensions construct from one to another. Values greater than 0.8 indicated acceptable reliability. The awareness domain constituted 38 statements with a Cronbach alpha of a = 0.820, the prevention domain had a = 0.97, the attitude domain had a = 0.73, and the perception domain had a = 0.97. Overall, this meant that all items were reliable, and sub-items were scale-labeled by each specific criterion. Therefore, this instrument maybe useful in measuring the awareness, attitudes, prevention, and perceptions of the COVID-19 outbreak among nurses.

Finally, four dimensions (awareness, prevention, attitude, and perception) having 62 items each were acknowledged. The item content validity index (I-CVI) ranged from 0.333 to 1, and the scale content validity index (S-CVI/Ave) ranged from 0.834 to 0.889. Therefore, the tool was measured with satisfactory content validity.

### 2.5. Data Analysis

The data was examined using Statistical Package for the Social Sciences (SPSS1) software (SPSS Inc., Chicago, IL, USA) version 24. To identify the association between nurse demographic characteristics and awareness, attitude, and perceptions of COVID-19, the Kruskal–Wallis test was applied, the Mann–Whitney test was used as a post hoc test, and the Shapiro–Wilk test was used.

## 3. Results

Table 1 represents the distribution of selected demographic data for this study; most participants (85%) were female nurses. The average age of the nurses was 33.92 (SD = 7.22). More than half of the nurses, 65%, were married, held bachelor’s degrees (66.8%), and were Saudi nurses (52.6%) working in the Eastern Region (Dammam, Jubail, and Hassa). Almost half of the nurses (45.6%) were working in a regional/public hospital. Before the COVID-19 outbreak, 25% of the nurses were only working 12 h/day, but that increased to 46.2% after the outbreak. More than half of the nurses (65.4%) received COVID-19 information from the MOH. Meanwhile, 28.8% of nurses were working in the emergency department, 14% in outpatient departments, and 6.8% in the operating room.

Table 2 describes nurses’ awareness, prevention, attitudes, and perceptions during the COVID-19 outbreak in Saudi Arabia. In terms of the awareness level of nurses about COVID-19, 96.85% had excellent awareness about the virus. In terms of preventive practice (awareness and skills) of nurses in dealing with COVID-19, 83.2% reported the highest prevention, while 38 (7.6%) had low prevention. More than half of respondents (60.4%) had a high positive attitude towards providing care to COVID-19 patients. Meanwhile, in terms of the perception of nurses towards COVID-19, more than half of the nurses (69.2%) had a very high perception.

As shown in Table 3, gender and nationality were abnormally distributed; therefore, the Mann–Whitney test was applied, and women respondents were statistically significantly higher than males in mean rank = 256, only for prevention, with U = 12,169.5 and a *p*-value = 0.02 for CI = 95%.

Marital status, educational attainment, region, hospital where employed, and area of practice were also abnormally distributed. Therefore, the Kruskal–Wallis test was applied and the Mann–Whitney test was applied as a post hoc test. Marital status showed a statistically significant difference between participants’ responses for the prevention and perception domains. There were statistically significant differences between responses for prevention, with K = 21.8 and a *p*-value = 0.00 for CI = 95%, and for the perception domain, K = 10.3 with a *p*-value = 0.016 for CI = 95%. The Mann–Whitney test was applied as a post hoc test to determine the source of statistically significant differences. Married respondents scored statistically significantly higher than single respondents in both the prevention and perception domains, with a mean rank = 253.3 and a *p*-value = 0.009 and a mean rank = 253.5 with a *p*-value = 0.005, respectively, and was also higher than divorced respondents, with a mean rank = 165.6 and a *p*-value = 0.000. Single respondents scored statistically significantly higher than divorced respondents for prevention only, with a mean rank = 99.5 and *p*-value = 0.001.

Education level showed a statistically significant difference in participants’ responses for the perception domain only. There was a statistically significant difference between participants’ responses for perception, with K = 10.02 and a *p*-value = 0.016 for CI = 95%; the Mann-Whitney test was applied as a post hoc test to determine the source of this difference. Diploma and BSN educated nurses scored statistically significantly higher than master’s educated nurses with mean rank = 88.02 and a *p*-value = 0.014, and a mean rank = 209.55 with a *p*-value = 0.010, respectively.

In the category of experience, there were statistically significant differences between participants in the attitude domain only, with K = 12.6 and a *p*-value = 0.013 for CI = 95%. The Mann–Whitney test was applied as a post hoc test to determine the source of this difference. The 21 to 30 years of experience group scored statistically significantly higher in experience than the less than 10 years group, and 11 to 20 years of experience group had a mean rank = 223.5 with a *p*-value = 0.010 and a mean rank = 109.63 with a *p*-value = 0.041, respectively.

Non-Saudi nurses were statistically significant compared to Saudi nurses in awareness, prevention, and perception, with a *p*-value less than 0.05 and a mean rank = 302.1, 272.1, and 283.69, respectively; while Saudi nurses were statistically significantly higher than non-Saudi nurses for attitude, with a mean rank = 265.6 and a *p*-value = 0.009.

The area of practice showed statistically significant differences between participants’ responses for the awareness and perception domains; for awareness, K = 16.5 with a *p*-value = 0.0054, and for perception, K = 14.72 with a *p*-value = 0.012. The Mann–Whitney test was applied as a post hoc test to determine the source of this difference. Operating room nurses were statistically significantly higher than the other departments, with a *p*-value of 0.005.

## 4. Discussion

The study examined nurses’ awareness, attitudes, and perceptions during the COVID-19 outbreak in Saudi Arabia. Due to limited literature pertaining to COVID-19, results were compared with other related coronaviruses, such as MERS-CoV and SARS-CoV. Several findings were highlighted in the study.

Based on the results, more than half of the nurses (65.4%) received their COVID-19 information from the MOH [14]. This is similar to other studies conducted in Saudi Arabia, in which 50% of the nurses received their health information from the MOH website about other coronavirus diseases such as MERS-CoV [20]. The MOH strives to increase accessibility to health materials and information delivery, with the aim to improve healthcare providers’ awareness, perception, and awareness (MOH, 2020) [14]. Aldohyan et al. [21] noted that healthcare workers from primary care settings in Saudi Arabia received information through the MOH website. MOH information is considered scientifically reliable, validated, up to date and similar to the mandates of WHO guidelines [2,3]. Previous studies reported that disease information from, for example, social media may not be scientifically valid [20].

One issue this study revealed is that 25% of the nurses worked 12 h/day before the COVID-19 outbreak. After the COVID pandemic began, that increased to 46.2%. This may be a result of increased hospital admission, variations in inpatient needs, or unforeseen understaffing. According to Thompson [22], nurses working longer than 12-hour shifts are at higher risk for fatigue and burnout, leading to compromised quality patient care. Banakhar [23] stated that nurses working for 12 h affects their well-being, and increases stress, fatigue, and anxiety compared to an 8 h shift. One study among European nurses working more than 12 h describes the quality of nursing care provided to patients on their unit as poor, assesses patient safety as fair, and accounts additional care instruments and equipment left uncompleted on their last shift in comparison with nurses working ≤ 8 h and no more than their contracted hours. Longer working hours might affect the efficiency and effectiveness of the workforce in delivering high-quality, safe care [24]. A recent study in China about healthcare providers working longer hours due to the spread of COVID-19 conveyed high symptom rates of depression, insomnia, and work stress [25]. An international study reported that when nurses wear personal protective equipment (PPE), they usually take 4–6 working hours without a break. This is very critical to nurses’ well-being, since longer hours wearing PPE can cause fatigue, stress, and exhaustion, making healthcare providers prone to causing medical errors [26]. Hence, nursing administration should organize staffing and scheduling to avoid mental and physical health impairment.

Important in this study is that nurses had awareness and had high positive perceptions about COVD-19. This could be because the MOH constantly updates its website and obligates healthcare providers to be aware of COVID-19 updates. It is worth noting that nurses working in Saudi have experience with other coronavirus diseases, such as MERS [27]. Lessons learned treating these infections may strengthen nurses’ ability to prepare, adapt, and effectively respond to any disease outbreak. Studies show nurses in other countries with a COVID-19 outbreak, such as Iran [28], Saudi Arabia [29], and Korea [30] with MERS-CoV, had higher perception and awareness levels. According to Aldohyan [21], insufficient awareness of infectious diseases could lead to inappropriate infection control and precaution measures. Thus, providing educational programs about COVID-19 could increase awareness and improve infection control and prevention. Nevertheless, caution should be used when interpreting the previous studies, since they used different tools and participants, which might affect awareness and perception levels. Further studies about awareness and perception using a validated tool are warranted.

Nurses in this study reported higher preventive practices in dealing with COVID-19. These findings affirm a previous study among healthcare workers on MERS-CoV in Saudi, and a recent study about COVID-19 in India among students and health care workers, in which, due to constant exposure and previous outbreak experience with similar coronavirus disease, the nurses were able to practice in their full clinical capacity and use preventive measures [31]. Better preventive practices in these findings could be elucidated by the fact that educational campaigns, particularly the MOH in Saudi Arabia, focused more on signs and symptoms of COVID-19, which may have enhanced preventive practices.

In establishing efficient methodologies to support healthcare professionals, it is vital to realize their attitudes as well as their specific sources of anxiety and fear. Ironically, the specific statement “I feel anxious about transmitting the infection to my family members” received the highest positive mean among the attitude statements. Anxiety about the infection has a pivotal role in prevention and management of infection, since it can weaken confidence in giving patient care [32]. Adams and Walls (2020) [33] reported that during an outbreak, nurses face bigger exposure risk, tremendous workloads, ethical dilemmas, and a speedily developing practice environment that varies importantly from what is familiar, thus affecting their attitudes toward infection prevention. During an infection outbreak, healthcare professionals experience negative emotions, like fear, anxiety, and helplessness, as seen in studies on COVID-19 in China [34], MERS-CoV in Korea [30], and Ebola in the US [35]. Recognizing the sources of these negative emotions—anxiety, burnout, fatigue, and stress—can improve attitudes and spur nursing administrators and organizations to improve directed methodologies in addressing these apprehensions and offer detailed support in the healthcare workforce.

In this study, nurses had a positive perception of COVID-19 management and prevention. This implies that nurses are confident in existing infection prevention and control programs. This also could be explained by the respondents’ view that they possessed greater awareness about prevention. This agrees with a previous study that showed a positive perception from healthcare workers when dealing with a MERS-CoV outbreak in Saudi Arabia [29]. Because MERS and COVID-19 have almost the same symptoms and route of transmission, nurses have familiarity with similar infections. The results are also in agreement with a Vietnam study pertaining to perception and attitude toward COVID-19 among healthcare professionals [36]. These previous studies found that a positive perception is related to high awareness and sufficient COVID-19 prevention control.

Interestingly, in this study, demographics and work-related issues mattered. Female nurses had better preventive behaviors than male nurses. This distinction can be attributed to the fact that, in Saudi tradition and culture, females are more inclined to be healthcare providers than men [37]. This result is consistent with a United Nations policy brief that women are more confident and have higher self-awareness about the impact of COVID-19 on women [38]. Caution should be taken in interpreting this study, since only 13.2% of participants were male nurses, which means the findings cannot be generalized.

Marital status significantly impacts prevention and perception domains on COVID-19. In this study, married nurses, compared to single nurses, scored statistically significantly higher in both the prevention and perception domains on COVID-19. This affirms previous empirical information that married nurses working in hospitals not only care for patients, but also interact and care for their families [39]. Therefore, married nurses might be extra careful when caring for COVID-19 patients to prevent transmitting the disease to their families. As a result, married nurses might have a strong motivator to study infectious diseases and be more protected against infection than single nurses. According to Condliffe, Link, & Zengin Farias Martinez [40], married women have families, so their preventive behavior is higher in response to working conditions, and they are more careful in dealing with a patient compared to single nurses. This means that social support from marriage has preventive effects on reducing infection risk. Furthermore, married nurses foster greater social relationships, emotional support, and economic security [23].

More empirical data reported that married people have a better attitude toward infectious diseases, are more likely to avoid risky behavior, and a higher percentage of seeing a doctor for checkups and screenings [24]. Hence, with several advantages of being a married individual, it is most likely that it could positively influence their perception of diseases as well as improve their attitude on how to prevent and protect from COVID-19.

It may be no surprise that nurses with a bachelor’s degree had better prevention and perception towards COVID-19 compared to other educational backgrounds. This is expected, since most of the nursing staff hold bachelor’s degree as a minimum requirement to work as a staff nurse. Bachelor’s degree nurses are accountable for early patient monitoring assessment and nurturing patients towards recovery, while nurses holding master’s degrees are in managerial and supervisory positions [41]. It is worth noting that bachelor’s degree nurses are accountable for and oversee the provision of safe patient care, thus with greater exposure and consistent care, it may expand their field of knowledge and skills of practice to provide for suitable outcomes for patients. This might be the reason why bachelor’s degree nurses have better prevention and perception towards COVID-19: because of more exposure in bedside care.

However, this negates the previous studies that nurses with master’s degrees have positive clinical roles, with higher-level clinical competence and more knowledge than bachelor’s degree nurses [42]. This is because they have advanced nursing practice skills learned during their academic courses in their master’s degree. Another previous study stated that nurses with a master’s degree made an optimistic influence on the quality of services and patient safety. Nurses who enrolled in a master’s degree program develop more of an ability to think critically, which could be reinforced in a master’s degree program [43]. This interpretation resembles a previous study showing that a master’s degree is expected to provide more training in critical and analytical thinking in comparison with a bachelor’s degree. [44]. Thus, this might enhance nurses’ expertise, allow them to gain new skills, and display more nursing core values and beliefs through their actions. These positive outcomes have resulted in explaining why nurses should enroll for further education. Nevertheless, this result commands further investigation that assesses the added value of a master’s degree compared to a bachelor’s degree in a Saudi Arabian healthcare context.

Additionally, work experience affects attitude towards COVID-19, according to this study. Specifically, nurses with 11 to 20 years’ experience have a better attitude than those with less than 10 years. It is worth considering that the longer the work experience, the better they are equipped in handling patients. It could also be safe to say that more work experience translates to increased awareness of COVID-19, better preventive skills, and improved patient care. Kieft, de Brouwer, Francke, and Delnoij [45] stated that the work experience nurses have, the more opportunity to practice critically and provide quality patient care. This might be interpreted as work experience can shape awareness, attitudes, perception, and preventive behavior, and create a strong nursing practice, resulting in more positive patient experiences.

Finally, non-Saudi nurses self-reported higher perception, prevention, and attitudes towards COVID-19 than Saudi nurses. This is expected, since most of the front-line nurses in the country are expatriates [46]. Most Saudi nurses occupy managerial and supervisory positions, with their main focus on staff management, recruitment, budgeting, scheduling, discharge planning, developing educational plans, and records management, but not direct patient care. This is something to address, because there is a Saudi policy to ensure Saudi nationals are prepared to fill important positions when non-Saudi nurses leave the country [37].

Finally, in this study, nurses working in operating rooms (ORs) have better awareness, prevention, and attitudes than any other participants. This is expected, since nurses working in the OR are trained and equipped with standard or enhanced PPE at all times. This is similar to a study in Singapore, wherein OR nurses are constantly attending seminars about implementing strict guidelines or references for COVID-19 prevention [47]. It could be safe to say that constant reinforcement of health information could lead to better awareness, prevention, and attitudes. As main providers of patient care before, during, and after surgery, OR nurses are required to have awareness and skills for preventing surgical site infection, patient risk assessment, environmental cleaning, disinfection, sterilization of instrumentation, patient antibiotic prophylaxis, and the use of standard precautions.

### Limitations

Some limitations should be considered in interpreting this study. The questionnaire is subject to recall bias and misclassification. Another limitation was the use of self-reported measures, convenience sampling, and use of a cross-sectional design, which cannot be generalized to the study findings. In addition, most of the respondents were young, married, held bachelor’s degrees, and were Saudi nurses. Therefore, generalization of the findings is impossible.

Working hours among nurses was not added in the association between nurses’ demographics information towards their awareness, attitude, and perceptions of the COVID-19 outbreak. It could be interesting to note for future research how nurses’ working hours affect their awareness, attitude, and perceptions of COVID-19. Further study, such as a longitudinal study design, could provide an analysis of cause and effect relationships between nurses’ demographic characteristics and awareness, positive attitudes, optimal prevention, and positive perceptions during the COVID-19 outbreak. It would also be interesting to further explore awareness, attitude, and prevention practice regarding other similar coronavirus diseases (e.g., SARS and MERS-CoV), since they have similar clinical presentations ranging from asymptomatic to acute respiratory distress syndrome, and multi-organ failure leading to death. However, this validation didn’t compute with current standards (analysis of the internal structure, evidence of external validity, and intra-observer reliability,) which limits the validity and reliability of the data. Therefore, exploration about the internal structure, evidence of external validity, and intra-observer reliability of the tool warrants further attention. One of the strengths of the study is that it was conducted during the COVID-19 outbreak, thus, the exact influence of the outbreak on nurses’ awareness, attitudes, prevention, and perceptions could be measured in real time.

## 5. Conclusions

This study highlighted that nurses had greater awareness, positive attitudes, optimal prevention, and positive perceptions during the COVID-19 outbreak in Saudi Arabia. Female nurses, married nurses, and bachelor’s degree nurses had better attitudes and prevention towards the virus. Meanwhile, non-Saudi nurses had higher self-reported awareness, positive attitudes, optimal prevention, and positive perceptions compared to Saudi nurses. Finally, the results provide a theoretical underpinning that expands on previous awareness and literature on factors affecting the awareness, attitudes, prevention, and perceptions about the COVID-19 outbreak.

## 6. Implications

This study aimed to assess the awareness, attitudes, prevention, and perceptions about the COVID-19 outbreak. Results were vital to advance awareness, perception, attitudes, and clinical practices in managing the COVID-19 patient. The study has significant implications for future actions, such as improving self-care by increasing COVID-19 prevention awareness and control guidelines. The study also summarizes key considerations for supporting the health care workforce, so nurses are equipped to provide care for their patients and communities. The study recommends that the MOH promote COVID-19 with a comprehensive training program during and after the outbreak to ensure scientifically proven clinical awareness. The MOH, WHO, and CDC websites should also be updated regularly. Finally, aside from COVID-19, there are likely to be other new communicable infections to deal with in the future. In this regard, a long-term policy, structures, and strategies are needed to strengthen healthcare systems and, specifically, the capacity and capability to respond properly to any future public health emergencies. It is noteworthy that, in order to achieve a sustainable healthcare system with knowledgeable and skilled nurses in infection management, advanced human resources planning is absolutely crucial.

## Figures and Tables

**Table 1:** Nurses’ demographic and work-related characteristics (*n* = 500).

Profile	Frequency	%	Mean	SD
Age			33.92	7.22
Gender				
Male	69	13.2		
Female	431	86.2		
Marital Status				
Single	175	35		
Married	325	65		
Educational Attainment				
Diploma nursing	89	17.8		
Bachelor’s degree graduate	334	66.8		
Master’s degree graduate	71	14.2		
PhD graduate	6	1.2		
Nationality				
Saudi	263	52.6		
Indian	121	24.2		
Filipino	111	22.2		
Egyptian	4	0.8		
Sudanese	1	0.2		
Saudi Region in which nurses are presently working				
Central Region (Riyadh, Qassim)	46	9.2		
Eastern Region (Dammam, Jubail, Hassa)	258	51.6		
Western Region (Makkah, Jeddah, Taif & Madinah)	113	22.6		
Northern Region (Hail, Aljouf, Tabouk & Arar)	21	4.2		
Southern Region (Assir, Jazan, Najran, Baha)	62	12.4		
Workplace				
Regional/Public Hospital	228	45.6		
Primary Healthcare Center	118	23.6		
Specialized Hospital	72	14.4		
University Hospital	11	2.2		
Military Hospital	50	10		
Private Hospital	21	4.2		
Area of Practice				
Outpatient Department	70	14		
Surgical ward	54	10.8		
Emergency room	144	28.8		
Operating Room	34	6.8		
Intensive Care Unit	99	19.8		
Medical ward	99	19.8		
Working hours per week before COVID-19 outbreak				
8 h	375	75		
12 h	125	25		
Working hours per week after the COVID-19 outbreak				
8 h	269	53.8		
12 h	231	46.2		
Main source of clinical information regarding COVID-19				
Saudi Ministry of Health	327	65.4		
World Health Organization	97	19.4		
Social Media/Public News	37	7.4		
Health Care Professional/Colleague	9	1.8		
Internet Resources	26	5.2		
Published Literature	4	0.8		

**Table 2:** Descriptive information of nurses’ awareness, prevention, attitudes, and perceptions during the COVID-19 outbreak in Saudi Arabia.

**Awareness Level of Nurses towards COVID-19 (*n* = 500)**		
Aware	5	1
Well aware	11	2.2
Fully aware	484	96.8
Cronbach’s Alpha	0.82	
Content validity index (I-CVI)	0.86	
**Prevention of Nurses of COVID-9 (*n* = 500)**		
**Level**	**Frequency**	**Percent**
Low Prevention	38	7.6
Average Prevention	4	0.8
High Prevention	42	8.4
Highest Prevention	416	83.2
Cronbach’s Alpha	0.972	
Content validity index (I-CVI)	0.88	
**Attitude of Nurses towards COVID-9 (*n* = 500)**		
**Level**	**Frequency**	**Percent**
Acceptable Attitude	55	11.0
Good Attitude	143	28.6
High Attitude	302	60.4
Cronbach’s Alpha	0.73	
Content validity index (I-CVI)	0.88	
**Perception of Nurses towards COVID-9 (*n* = 500)**		
**Level**	**Frequency**	**Percent**
Low Perception	10	2.0
Average Perception	41	8.2
High Perception	103	20.6
Very High Perception	346	69.2
Cronbach’s Alpha	0.97	
Content validity index (I-CVI)	0.88	

**Table 3:** Association between nurses’ demographic information and their awareness, attitude, and perceptions of the COVID-19 outbreak (*n* = 500).

Demographic Information		Known			Prevention			Attitude			Perception		
		Mean	Std. Deviation	*p*-Value	Mean	Std. Deviation	*p*-Value	Mean	Std. Deviation	*p*-Value	Mean	Std. Deviation	*p*-Value
Gender	Female	1.87	0.12	0.08	4.33	0.94	0.02	2.59	0.45	0.94	2.57	0.52	0.59
	Male	1.82	0.17		3.95	1.26		2.6	0.44		2.57	0.57	
Age Group	from 18 to less than 27 years	1.87	0.08	0.76	4.21	1.01	0.23	2.55	0.46	0.27	2.67	0.43	0.03
	from 27 to less than 36 years	1.86	0.12		4.25	1.03		2.59	0.45		2.51	0.55	
	from 36 to less than 45 years	1.85	0.17		4.32	0.99		2.65	0.44		2.61	0.53	
	from 45 to less than 54 years	1.88	0.09		4.54	0.71		2.67	0.42		2.68	0.48	
	from 54 to less than 63 years	1.79	0.28		3.96	1.36		2.39	0.57		2.46	0.78	
Marital Status	Single	1.86	0.11	0.154	4.18	1.06	0	2.59	0.43	0.926	2.509	0.515	0.016
	Married	1.86	0.13		4.41	0.85		2.6	0.46		2.615	0.524	
	Divorced	1.79	0.19		2.6	1.61		2.56	0.57		2.417	0.556	
	Widowed	1.91	0.05		4.63	0.41		2.75	0.32		2.750	0.5	
Educational Attainment	Diploma	1.821	0.205	0.391	4.201	0.974	0.148	2.607	0.451	0.482	2.598	0.587	0.018
	BSN	1.874	0.093		4.310	0.98		2.595	0.454		2.597	0.49	
	Master’s	1.860	0.112		4.244	1.095		2.563	0.435		2.408	0.581	
	PhD	1.746	0.254		3.821	1.408		2.833	0.279		2.833	0.258	
Experience Groups	Less than 10 years	18.655	0.09171	0.545	43.006	0.94013	0.176	25.556	0.45808	0.013	25.665	0.49736	0.443
	From 11 to 20 years	18.522	0.16941		41.753	114.564		26.320	0.44144		25.649	0.57152	
	From 21 to 30 years	18.569	0.18256		44.710	0.79495		28.125	0.31609		26.719	0.55153	
Nationality	Saudi	1.825	0.154	0	4.148	1.102	0.001	2.665	0.372	0.009	2.455	0.568	0
	Non-Saudi	1.900	0.07		4.417	0.854		2.518	0.51		2.704	0.434	
Region	Central Region (Riyadh, Qassim)	1.820	0.184	0.139	4.006	1.235	0.122	2.601	0.437	0.15	2.527	0.533	0.341
	Eastern Region (Dammam, Jubail, Hassa, and other)	1.864	0.122		4.356	0.932		2.642	0.41		2.549	0.536	
	Western Region (Makkah, Jeddah, Taif, and Madinah)	1.883	0.09		4.292	0.942		2.584	0.46		2.577	0.53	
	Northern Region (Hail, Aljouf, Tabouk, and Arar)	1.826	0.162		3.854	1.321		2.508	0.512		2.595	0.497	
	Southern Region (Assir, Jazan, Najran, and Baha)	1.851	0.136		4.253	1.028		2.446	0.536		2.690	0.454	
Worked Hospital	Regional/Public Hospital	1.869	0.083	0.161	4.246	1.057	0.976	2.588	0.459	0.995	2.564	0.552	0.314
	Primary Healthcare Center	1.832	0.19		4.306	0.929		2.588	0.45		2.574	0.519	
	Specialized Hospital	1.853	0.136		4.375	0.724		2.602	0.439		2.642	0.44	
	University Hospital	1.809	0.209		4.331	1.027		2.545	0.583		2.318	0.767	
	Military Hospital	1.894	0.067		4.083	1.345		2.627	0.396		2.505	0.459	
	Private Hospital	1.907	0.062		4.524	0.458		2.651	0.441		2.726	0.467	
Area of Practice	Outpatient Department	1.810	0.167	0.005	4.363	0.934	0.589	2.590	0.422	0.536	2.461	0.57	0.012
	Surgical Department	1.886	0.067		4.339	0.762		2.574	0.516		2.667	0.461	
	Emergency Department	1.868	0.129		4.124	1.207		2.634	0.46		2.578	0.48	
	Operating Room	1.887	0.081		4.492	0.733		2.657	0.312		2.426	0.569	
	Intensive Care Unit	1.878	0.1		4.338	0.867		2.542	0.446		2.677	0.522	
	Medical Department	1.848	0.145		4.264	1.023		2.586	0.457		2.540	0.548	

## Supplementary Materials

The following are available online at https://www.mdpi.com/1660-4601/17/21/8269/s1.
