# Supplementary material for: Awareness, Attitudes, Prevention, and Perceptions of COVID-19 Outbreak among Nurses in Saudi Arabia
Source: Int J Environ Res Public Health. 2020 Nov 9;17(21):8269. doi: 10.3390/ijerph17218269 (PMC7664870; doi:10.3390/ijerph17218269)
Supplement: Supplementary file 1 [file ijerph-17-08269-s001.pdf]

Awareness, Attitude and Perceptions of COVID-19 Outbreak among Health Care Providers in Saudi Arabia

Dear Health Care Provider,
You are invited to participate in a study entitled "Awareness, Attitude and Perceptions of COVID-19 Outbreak among Health Care Provider in Saudi Arabia". This questionnaire intends to assess the health care provider's awareness about the pandemic in terms of the causative virus, signs and symptoms, transmission, and management protocol. In addition, complications, preventive methods, attitudes, perceptions and practices regarding the COVID-19.

This questionnaire takes about twenty minutes to complete. All your answers will be treated with strictest confidence. Your participation in this study is voluntary and you may withdraw from the study at any time.
Thank you in advance for your participation

PART I. Demographic and work-related characteristics.

1. **Age**: _______________

2. **Gender**
   [ ] Male
   [ ] Female

3. **Marital status**
   [ ] Single
   [ ] Married
   [ ] Divorced
   [ ] Widowed

4. **Educational attainment**

   [ ] Diploma
   [ ] Bachelor
   [ ] Master's
   [ ] PhD
   [ ] Others specify _______________

5. **Nationality**
   [ ] Saudi
   [ ] Filipino
   [ ] Indian
   [ ] Egyptian
   [ ] Sudanese
   [ ] Others specify _______________

6. **Total years of clinical experience as a health care provider: _______________**

7. **Region you work at**

[ ] Central Region (Riyadh, Qassim)
[ ] Eastern Region (Dammam, Jubail, Hassa & others)
[ ] Western Region (Makkah, Jeddah,Taif & Madinah)
[ ] Northern Region (Hail, Aljouf, Tabouk & Arar)
[ ] Southern Region (Assir, Jazan, Najran, Baha)

8. **Your Workplace:**

[ ] Primary Healthcare Center
[ ] Regional / Public Hospital
[ ] Specialized Hospital
[ ] Private Hospital
[ ] University Hospital
[ ] Military Hospital
[ ] Others specify _______________

9. **Specialty**

[ ] Physician
[ ] Nurse
[ ] Dentist
[ ] Pharmacists
[ ] Laboratory Specialist
[ ] Physical therapists
[ ] Others specify _______________

10. **Working hours per day before COVID-19 outbreak:**

[ ] 8 hrs
[ ] 12 hrs
[ ] Others specify _______________

11. **Working hours per day after the COVID-19 outbreak:**

[ ] 8 hrs
[ ] 12 hrs
[ ] Others specify _______________

12. **What is your main source of clinical information in regards to COVID-19?**

[ ] Saudi Ministry of Health
[ ] World Health Organization
[ ] Social Media/Public news
[ ]] Health Care Professional/ Colleague

[ ] Published literature
[ ] Internet resources _______________
[ ] Others specify _______________

Part II. Awareness of COVID-19.
Put a check mark on the box that best describes your opinion about each statement. Base your answers on the following scale:
1 – Strongly Disagree
2 – Disagree
3 – Don't know/ Neutral
4 – Agree
5 – Strongly Agree

No	Statement	1	2	3	4	5
1	COVID -19 belongs to the RNA viruses family					
2	COVID -19 is also known as SARS CoV-2					
3	COVID -19 originated from bats and then it was transmitted to other mammalian hosts					
4	COVID -19 is transmitted by close contact with infected individuals.					
5	COVID -19 is transmitted by close contact with infected animals.					
6	COVID -19 transmission is believed to occur through respiratory droplets from coughing and sneezing.					
7	Individuals who have been exposed to COVID -19 may transmit the infection even before they have symptoms.					
8	COVID -19 can be transmitted from sharing personal items.					
9	COVID -19 incubation time could generally be within 3 to 7 days and up to 2 weeks.					
10	The symptoms of COVID -19 are fever, dry cough, and difficulty breathing.					
11	Individuals with co-morbidity (diabetes mellitus and other chronic diseases) are more likely to be infected with COVID -19 compared to healthy ones.					
12	Individuals with co-morbidity (diabetes mellitus and other chronic diseases) are more likely die from COVID -19 than healthy ones.					
13	COVID -19 has less fatality rate than MERS –CoV.					
14	COVID -19 can be transmitted by talking to any individuals with distance less than one meter.					
15	COVID -19 can be transmitted through handshaking and hugging.					
16	COVID -19 can be transmitted through touching your face, eyes, nose and mouth.					

17	COVID -19 can be transmitted through touching infected surfaces (door handles. stair poles, equipment).					
18	COVID -19 can be transmitted through being in a social crowding.					
19	COVID -19 can be transmitted through eating raw or undercooked meat or animal organs.					
20	Complications of COVID -19 may include Pneumonia					
21	Complications of COVID -19 may include multiple organ failure.					
22	Complications of COVID -19 may include death.					
23	No vaccine is currently available against COVID -19.					
24	A patient diagnosed with COVID -19 can be cured.					
25	Individuals may test positive for COVID -19 ven when they have no signs or symptoms of the infection.					
26	Current therapeutic strategies to deal with the COVID -19 infection are only preventive and supportive.					
27	Isolation measures are so far the best approach to contain COVID -19 infection					
28	Health care providers must follow infection control measures with all individuals (health care providers and clients) regardless of whether they have symptoms or not.					
29	Individuals who have had a close contact with a COVID-19 confirmed patient positive for within 14 days before symptom onset must be quarantined and tested.					
30	Individuals who have a history of travel to countries that have a spread out of infection in the past 14 days should be quarantined and tested					
31	The protocol based on the WHO recommendation of collecting specimens are from both the upper respiratory tract (naso- and oropharyngeal samples) and lower respiratory tract such as expectorated sputum, endotracheal aspirate:<br>1. Bronchoalveolar lavage fluid<br>2. Sputum<br>3. Nasal swab<br>4. Fibrobronchoscope brush biopsy<br>5. Pharyngeal swab					
32	If the test result is positive, it is recommended that the test is repeated for verification.					
33	There is no specific antiviral treatment recommended for COVID-19.					
34	Oxygen therapy is the mainstay intervention for patients with severe infection.					
35	Mechanical ventilation may be necessary in cases of respiratory failure.					
36	Hemodynamic support is essential for managing septic shock.					

37	Health care provider should take special precautions during intubation by using personal protective equipment (PPE) such as FFP3 or N95 mask, protective goggles, disposable gown long sleeve raincoat, disposable double socks, and gloves.					
38	Most of the fatalities have occurred in patients over 50 years of age.					

Part III. Prevention of COVID-19.

Put a check mark on the box that best describes your opinion about each statement. Base your answers on the following scale:

1 – Strongly Disagree
2 – Disagree
3 – Don't know/ Neutral
4 – Agree
5 – Strongly Agree

No	Statement	1	2	3	4	5
1	Health care provider should keep their knowledge updated about COVID-19 from trustworthy sources such as the Saudi Ministry of Health and the WHO.					
2	Prevalence of COVID-19 can be reduced by active participation of health care providers in hospital infection control program.					
3	Prevalence of COVID-19 can be reduced by strict application of precaution methods such as hand washing, avoid sharing personal items, using PPE, etc.					
4	Transmission of COVID-19 infection can be prevented using universal precautions proposed by WHO.					
5	Using N95 masks by health care providers when caring for a Corona patient is critically important.					
6	During the COVID -19 outbreak, every patient coming to the hospital should be considered infectious and all standard protocol should be followed.					
7	Health care providerd caring for infected individuals (COVID-19 patients') should follow contact and airborne precautions which include personal protective equipments (PPEs) such as N95 or FFP3 masks, eye protection, gowns, and gloves to prevent transmission of the pathogen.					
8	People with fever, cough and difficulty breathing should seek medical attention without delay.					
9	It is important to stay more than 1 meter (3 feet) away from others.					
10	Only suspected cases of COVID-19 patients should be kept in quarantine.					

11	Preventive strategies are focused on the isolation of patients and careful employment of infection control measures.					
12	Droplet, contact, and airborne precautions should be taken during specimen collection, and sputum induction should be avoided.					
13	I follow the WHO general recommendations for prevention of COVID-19 infection in my practice.					
14	I educate my patients about the WHO general recommendations for prevention of COVID-19 infection.					

Part IV. Attitude
Put a check mark on the box that best describes your opinion about each statement. Base your answers on the following scale:
1 – Strongly Disagree
2 – Disagree
3 – Don't know/ Neutral
4 – Agree
5 – Strongly Agree

No	Statement	1	2	3	4	5
1	I am willing to provide care to COVID-19 diagnosed patients.					
2	I feel anxious about acquiring the infection while providing care to COVID-19 diagnosed patients.					
3	I feel anxious about transmitting the infection to my family members.					
4	I feel anxious when there is lack of proper masks, gowns and eye gear which is imperiling the ability to protect myself from COVID-19.					
5	I feel anxious when my colleagues might acquire the COVID-19 and transmitting to others, affecting nurse's workload, and patient safety					
6	Having the infection compensation decrease my anxiety regarding acquiring the infection.					

Part V. Perception

Put a check mark on the box that best describes your opinion about each statement. Base your answers on the following scale:
1 – Strongly Disagree

2 – Disagree
3 – Don't know/ Neutral
4 – Agree
5 – Strongly Agree

No	Statement	1	2	3	4	5
1	In my perception the hospital that I work at has a clear protocol for managing COVID-19.					
2	In my perception I have enough knowledge about COVID-19.					
3	In my perception the I have enough clinical experience that enables me to provide care for COVID-19 patients with confidence.					
4	In my perception the hospital that I work at has provided me and my colleagues with enough training to deal with COVID-19 patients.					

If you have any questions of further queries you can contact the Principle Investigator Dr. Reem Al-Dossary via email: rnaldosari@iau.edu.sa
Imam Abdulrahman Bin Fasial University, Damamm
Saudi Arabia

-The end-

Thank you
